# Analysis of the respiratory component of heart rate variability in the Cururu toad *Rhinella schneideri*

**DOI:** 10.1038/s41598-017-16350-0

**Published:** 2017-11-23

**Authors:** Lucas A. Zena, Cléo A. C. Leite, Leonardo S. Longhini, Daniel P. M. Dias, Glauber S. F. da Silva, Lynn K. Hartzler, Luciane H. Gargaglioni, Kênia C. Bícego

**Affiliations:** 1 Department of Animal Morphology and Physiology, College of Agricultural and Veterinary Sciences, São Paulo State University, 14884-900 Jaboticabal, SP Brazil; 2 Department of Physiological Sciences, Federal University of São Carlos, São Carlos, SP Brazil; 3 Department of Biological Sciences, Wright State University, Dayton, OH USA; 4 National Institute of Science and Technology in Comparative Physiology (INCT Fisiologia Comparada), Jaboticabal, SP Brazil; 5 Department of Physiology, Ribeirão Preto Medical School, University of São Paulo, Ribeirão Preto, SP Brazil

## Abstract

Beat-to-beat variation in heart rate (*f*_*H*_) has been used as a tool for elucidating the balance between sympathetic and parasympathetic modulation of the heart. A portion of the temporal changes in *f*_*H*_ is evidenced by a respiratory influence (cardiorespiratory interaction) on heart rate variability (HRV) with heartbeats increasing and decreasing within a respiratory cycle. Nevertheless, little is known about respiratory effects on HRV in lower vertebrates. By using frequency domain analysis, we provide the first evidence of a ventilatory component in HRV similar to mammalian respiratory sinus arrhythmia in an amphibian, the toad *Rhinella schneideri*. Increases in the heartbeats arose synchronously with each lung inflation cycle, an intermittent breathing pattern comprised of a series of successive lung inflations. A well-marked peak in the HRV signal matching lung inflation cycle was verified in toads whenever lung inflation cycles exhibit a regular rhythm. The cardiac beat-to-beat variation evoked at the moment of lung inflation accounts for both vagal and sympathetic influences. This cardiorespiratory interaction may arise from interactions between central and peripheral feedback mechanisms governing cardiorespiratory control and may underlie important cardiorespiratory adjustments for gas exchange improvement especially under extreme conditions like low oxygen availability.

## Introduction

The modulatory activity of the sympathetic and parasympathetic nervous system on the heart causes instantaneous heart rate (*f*_*H*_) oscillations on a beat-to-beat basis, the so-called heart rate variability (HRV). The respiratory and cardiovascular systems are coupled, reflecting a reciprocal interaction between the autonomic nervous system and respiratory control^1–3^. In mammals, within a respiratory cycle, *f*_*H*_ increases rapidly during inspiration then slows down during the expiratory phase, a cardiorespiratory-coupled phenomenon that is mediated by inspiratory inhibition of vagal tone on the heart^4,5^. This beat-to-beat variability in *f*_*H*_ can be assessed by examining their distribution in the frequency domain using power spectral analysis^6^. A high-frequency component in the spectrum of the HRV matches a peak at the respiratory frequency that is termed respiratory sinus arrhythmia (RSA)^6^. Although the underlying physiological relevance of the RSA is not completely understood, the prevailing hypothesis for its occurrence relates to improved pulmonary gas exchange effectiveness in the lungs by matching pulmonary perfusion with ventilation during inspiration^7^.

The cardiorespiratory interactions are very well described for mammals, but only sporadically studied in other vertebrate groups^8^. This is surprising given that there are striking examples of strong cardiorespiratory interactions across vertebrates. Among non-avian reptiles, for example, the freshwater turtle *Trachemys scripta* exhibits an episodic breathing pattern that is synchronized with a large and sustained elevation of *f*_*H*_ and pulmonary blood flow (two- to four-fold) that remains increased throughout the whole breathing episode resulting in a typical bradycardia during long breath-hold diving behavior^9–11^. Furthermore, a smaller magnitude tachycardia is synchronous with ventilation in the tortoise *Testudo graeca* and South American rattlesnake *Crotalus durissus terrificus*, and that is apparently more evident in species with a more continuous breathing pattern and shorter non-ventilatory periods^10,12^.

In anuran amphibians typically three distinct types of ventilatory behaviors can be recognized, namely buccal oscillations, lung ventilations and lung inflation cycles (see^3^ for review). Lung inflation cycles feature an intermittent episodic breathing pattern that commences with a series of exhalations followed by a series of lung inflations with no associated expiratory phase such that the lungs are progressively inflated^3,13,14^. In resting toads breathing normoxic air, ventilation consists of a variable number of breaths irregularly interspersed between each other, but becomes remarkably consistent during hypoxia which evokes lung inflation cycles in regular and brief intervals^13,15,16^. A possible cardiorespiratory interaction exists in anuran amphibians because *f*_*H*_ and pulmocutaneous blood flow (${\dot{Q}}_{{pc}}$) increase coincidently with the onset of the lung inflation cycle^3^. Therefore, our hypothesis is that the mechanism for cardiorespiratory interaction is present in anurans, but that coupling is only evident when the breathing pattern presents as a more regular rhythm^8^.

Analysis of HRV has been used as a powerful, well-established tool for elucidating the balance between sympathetic and parasympathetic modulation of *f*_*H*_. Measurements of the cyclic variations of *f*_*H*_ in a beat-to-beat basis using power spectral analysis in order to establish the autonomic balance and the relationship between respiratory and cardiovascular system has never been reported for a terrestrial amphibian. Both sympathetic and parasympathetic nervous systems influence *f*_*H*_ and ${\dot{Q}}_{{pc}}$ in anuran amphibians^3,17,18^. The extent to which the balance between vagal and sympathetic nervous systems accounts for augmented *f*_*H*_ and ${\dot{Q}}_{{pc}}$ within each lung inflation cycle of adult amphibians is not completely understood^3,19^. Therefore, the main objective of our study was to assess the presence of oscillatory components in the HRV signal evoked by lung inflation in the Cururu toad *Rhinella schneideri*. Variation between normoxia and hypoxia conditions (fraction of oxygen in the inspired air; F_I_O_2_ = 0.05) was used to assess alterations in cardiorespiratory interactions. HRV was assessed in time and frequency domains to evaluate the degree of variability in autonomic control of the heart. In order to assess the dimension of sympathetic and parasympathetic contribution to HRV, time-frequency analysis was performed before and after reversed selective autonomic blockade with a β-adrenergic receptor antagonist (*i*.*e*. sotalol hydrochloride) and a muscarinic receptors antagonist (*i*.*e*. atropine sulfate).

## Results

### Normoxic condition

**Figure 1:**
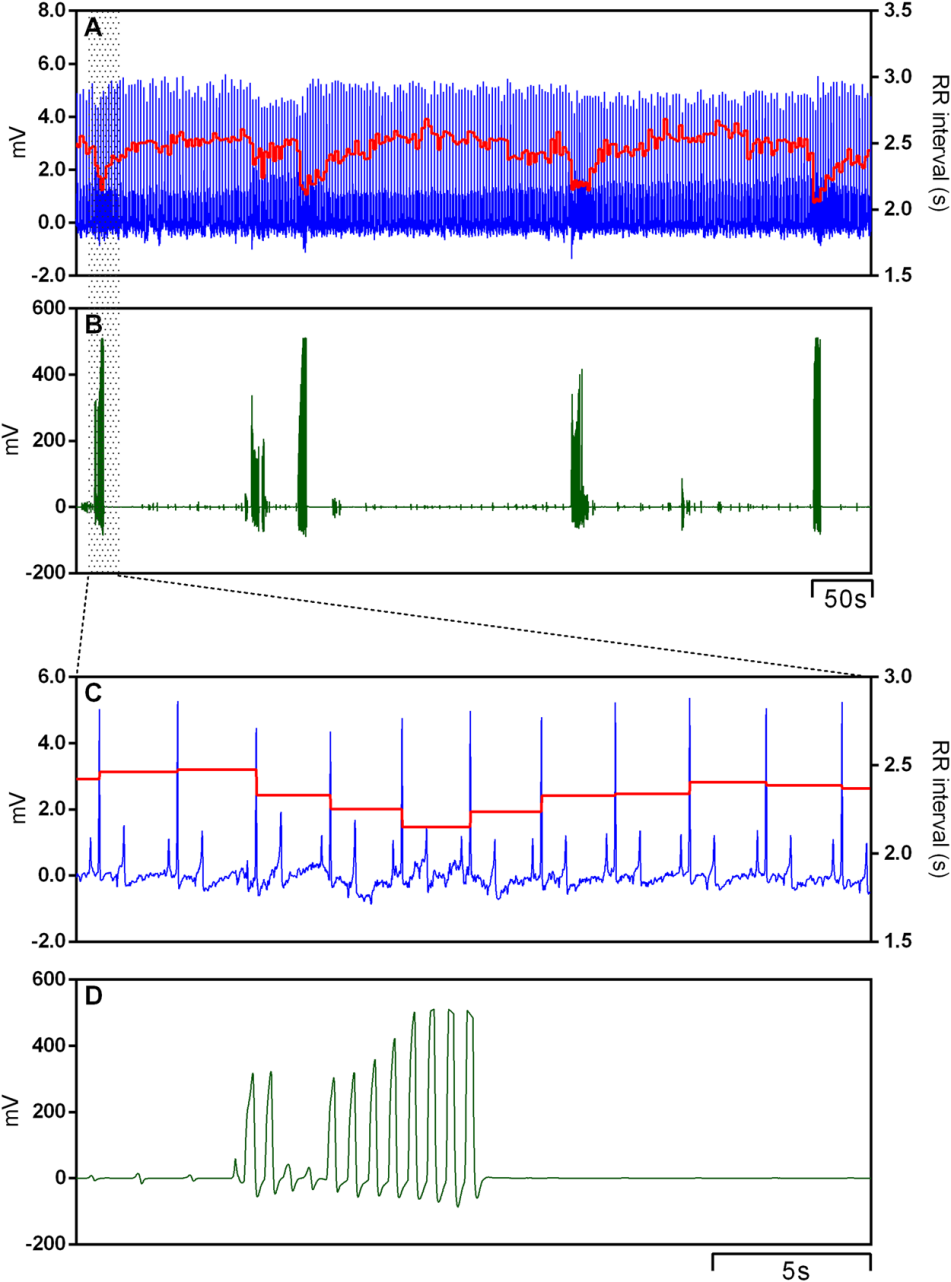
Representative traces showing raw electrocardiogram signal (ECG), the instantaneous RR interval **(A**) and the associated lung inflation cycles (buccal pressure cannula; **B**) in the cardiorespiratory interaction of one toad *Rhinella schneideri* during normoxia. High-speed traces show the ECG waveform (**C**) in pace with one lung inflation cycle (**D**).

**Figure 2:**
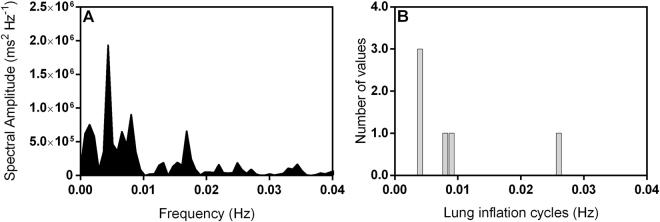
Representative power spectrum of the RR interval and associated histogram for lung inflation cycles in one toad *Rhinella schneideri* during normoxia. Spectrum of the RR interval exhibits a less defined ventilatory-related peak that emerges in a more scattered range of frequencies (**A**). Lung inflation cycles presented as frequency distribution histograms binned into 0.001 Hz width band (**B**) for comparison purposes with the spectrum of the RR interval (Data represents animal from Fig. 1).

**Figure 3:**
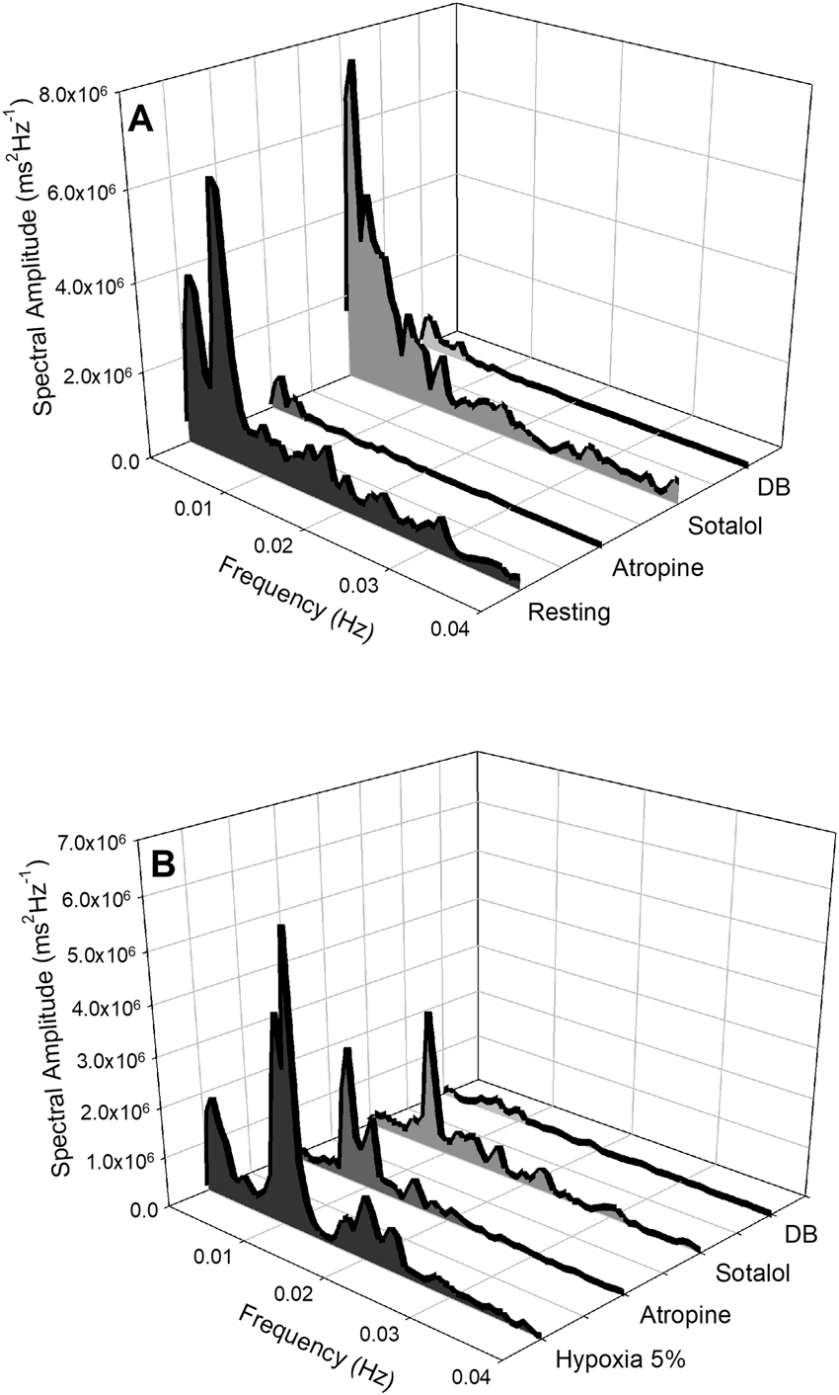
Waterfalls showing the spectra calculated under normoxia (**A**) and hypoxia (**B**) before and after selective autonomic blockade. Data sets consisting of 1,500 seconds were chosen within each condition: (**A**) Normoxia (*N* = 9); muscarinic blockade with atropine (*N* = 5); β-adrenergic blockade with sotalol (*N* = 4); and double autonomic blockade (DB; *N* = 9). (**B**) Hypoxia (F_I_O_2_: 0.05; *N* = 13); muscarinic blockade (*N* = 6); β-adrenergic blockade (*N* = 7); double autonomic blockade (DB; *N* = 13). Each spectrum was obtained following calculations of the average among all spectra of animals from the group at particular conditions. The standard error of the mean for the pooled data is omitted for clarity. For significant differences, see Table 1.

An example of cardiorespiratory interaction for one toad under normoxic conditions is depicted in Fig. 1 exhibiting a raw ECG recording overlapped by the instantaneous RR interval matching lung ventilation cycles. The 25-minute representative recording shows 5 lung inflation cycles synchronous with shortening of the RR interval that varies in magnitude. Although the power spectrum of the RR interval shows a ventilatory-related peak (Fig. 2), these emerge in a more scattered range of frequencies. The overall fluctuation in the R-R interval under normoxia was mostly abolished by muscarinic blockade: RR interval was reduced after muscarinic blockade (*F*_(3,24)_ = 11.494; *P* = 0.002), but remained unchanged after β-adrenergic blockade (*P* = 0.26) showing the predominance of parasympathetic modulation on the heart (Table 1; Fig. S1; Supplementary information). This is also corroborated by data of averaged TP_RR_ that show most of the HRV diminished by muscarinic (*F*_(3,24)_ = 37.474; *P* < 0.001), but not by β-adrenergic blockade (*P* = 0.71; Fig. 3A and Table 1). SD_RR_ was also significantly reduced after muscarinic blockade showing the prevailing effect of vagal modulation of heartbeat variability (*F*_(3,24)_ = 59.431; *P* < 0.001; Table 1).

**Table 1:** Cardiorespiratory parameters obtained during normoxia and hypoxia (F_I_O_2_: 0.05). The selective autonomic blockade with atropine (muscarinic blockade), sotalol (β-adrenergic blockade) and double autonomic blockade were performed under normoxic and hypoxic conditions in the Cururu toad *Rhinella schneideri* at 25 °C.

	Normoxia (*N* = 9)	Atropine (*N* = 5)	Sotalol (*N* = 4)	Double blockade (*N* = 9)
RR interval (s)	3.51 ± 0.26^a^	2.63 ± 0.17^b^	3.48 ± 0.52^a,b^	2.95 ± 0.19^b^
SD_RR_ (s)	0.29 ± 0.04^a^	0.06 ± 0.01^b^	0.24 ± 0.03^a^	0.05 ± 0.01^b^
TP_RR_ (ms^2^)	65449 ± 14793^a^	3419 ± 1995^b^	62107 ± 24404^a^	3337 ± 1991^b^
	**Hypoxia (F**_**I**_ **O**_**2**_ **:0.05) (** ***N*** ** = 13)**	**Atropine (** ***N*** ** = 6)**	**Sotalol (** ***N*** ** = 7)**	**Double blockade (** ***N*** ** = 13)**
RR interval (s)	1.74 ± 0.13^a^	1.74 ± 0.13^a,c^	2.25 ± 0.23^b^	2.02 ± 0.14^b,c^
SD_RR_ (s)	0.14 ± 0.04^a^	0.10 ± 0.03^a,b^	0.09 ± 0.03^a,b^	0.05 ± 0.01^b^
TP_RR_ (ms^2^)	18849 ± 8448^a^	7037 ± 4148^a,b^	5977 ± 3661^a^	1081 ± 431.2^b^
Lung inflation cycles (events.min^−1^)	0.93 ± 0.10	0.57 ± 0.06	1.00 ± 0.17	0.83 ± 0.17

SD_RR_, standard deviation of the RR intervals; TP_RR_, total power of the spectrum of the RR intervals; F_I_O_2_, fraction of oxygen in the inspired air.

Data are expressed as means ± s.e.m. Different letters indicate significant difference between treatments.

### Hypoxia

Toads inspiring hypoxic air (F_I_O_2_ = 0.05) exhibited a respiratory related-peak (0.01 Hz) in the spectrum of the RR intervals (Fig. 3B). Although isolated effects of either muscarinic or β-adrenergic blockade were not sufficient to significantly diminish the respiratory component (peak), their combined effects almost abolished the respiratory-related peak (Fig. 3B and Table 1; *P* < 0.001). Episodes of lung inflation cycles appeared in more regular time intervals during hypoxia, and occurred in a synchronized fashion with the shortening of the RR interval (increases in *f*_*H*_) within the lung inflation cycle episode followed by the lengthening of the RR interval once lung inflation ceased. The spectrum of the RR intervals reveals a fundamental peak emerging in a broader spectral range of frequencies (from 0.006 to 0.02 Hz; Fig. S2; Supplementary information), always matching lung inflation cycles frequency according to the respiratory response of the animal to hypoxia. The β-adrenergic blockade increased the RR interval in hypoxic conditions (*F*_(3,35)_ = 6.550; *P* = 0.003, Table 1), but muscarinic blockade had no effect on the RR interval (*P* = 1.0; Table 1). Collectively, double autonomic blockade significantly increased the RR interval under hypoxia mainly by affecting sympathetic modulation on the heart (*F*_(3,35)_ = 6.550; *P* = 0.032, Table 1).

**Figure 4:**
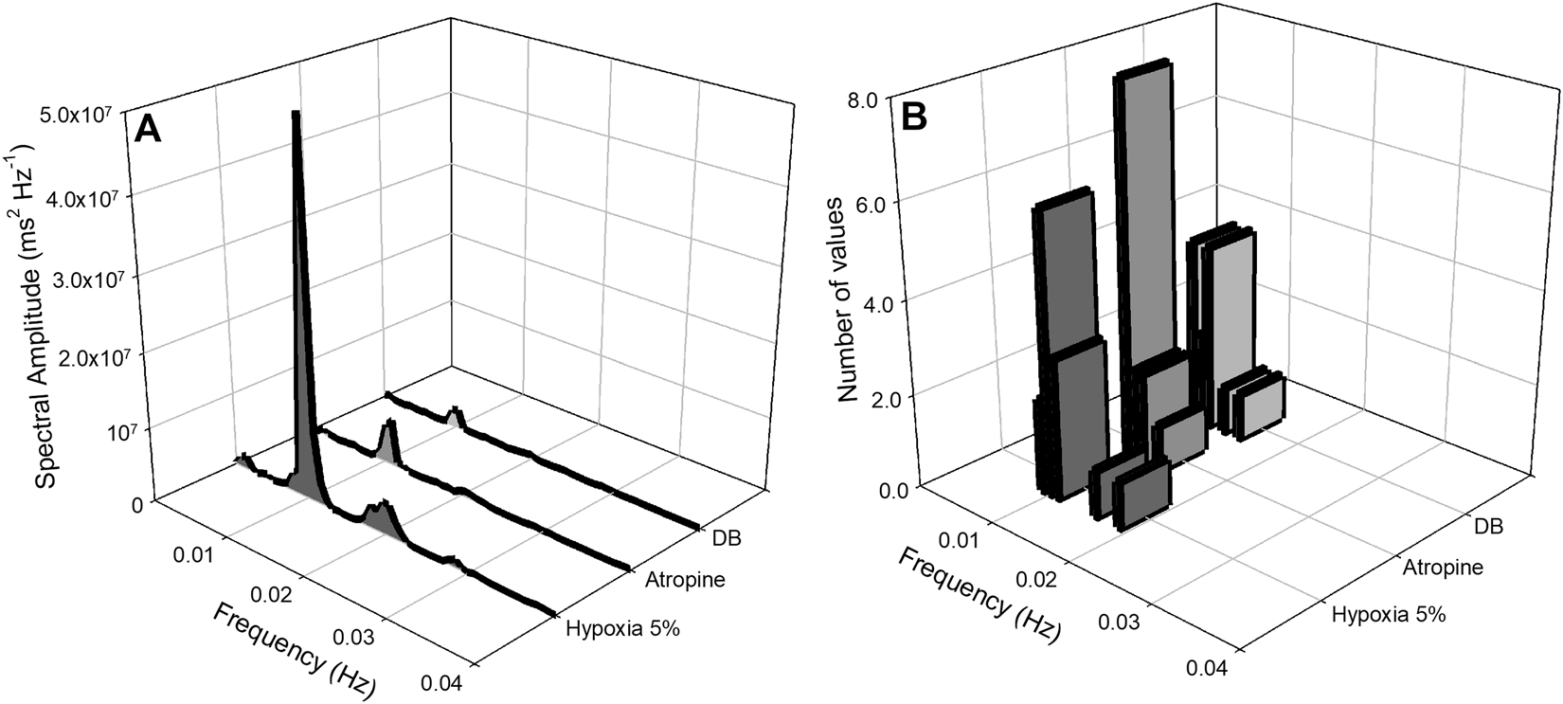
Cardiorespiratory interaction under hypoxia in the toad *Rhinella schneideri*. Waterfalls showing the spectra calculated (**A**) and associated histograms for lung inflation cycles (**B**) in one representative toad before and after selective autonomic blockade (atropine + sotalol) under hypoxic conditions (F_I_O_2_: 0.05). The respiratory-related peak in the heart rate variability was mostly abolished by the parasympathetic blockade (atropine). β-adrenergic after muscarinic blockade further diminished, but not eliminated, the remaining respiratory peak.

**Figure 5:**
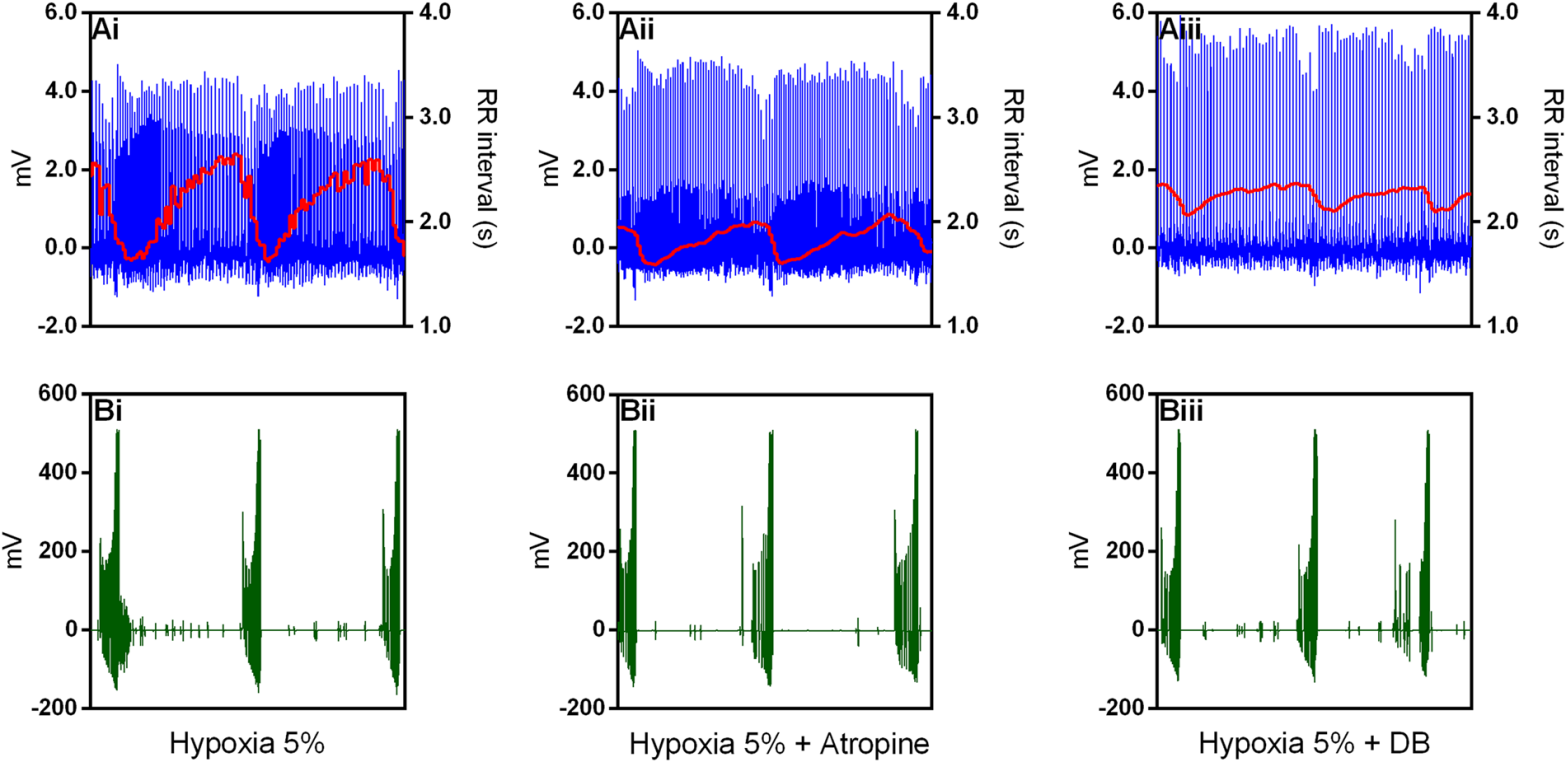
Representative recordings from one toad *Rhinella schneideri* (same as in Fig. 4) depicting raw electrocardiogram signals, the instantaneous RR interval and the associated lung inflation cycles (buccal pressure cannula) showing a cardiorespiratory interaction during hypoxia before (Ai and Bi), after muscarinic (Atropine; Aii and Bii) and double autonomic blockade (DB) (Aiii and Biii). Note that the amplitude oscillations in the RR interval associated with respiratory cycles were considerably diminished by the parasympathetic blockade (atropine).

**Figure 6:**
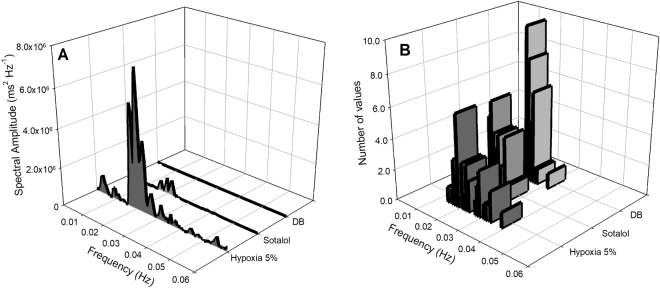
Cardiorespiratory interaction under hypoxia in the toad *Rhinella schneideri*. Waterfalls showing the spectra calculated (**A**) and associated histograms for lung inflation cycles (**B**) in one representative toad before and after selective autonomic blockade (sotalol + atropine) under hypoxic conditions (F_I_O_2_: 0.05). In contrast to Fig. 4, the respiratory-related peak in the heart rate variability was mostly abolished by the sympathetic blockade (sotalol). Muscarinic after β-adrenergic blockade completely abolished the remaining respiratory peak.

**Figure 7:**
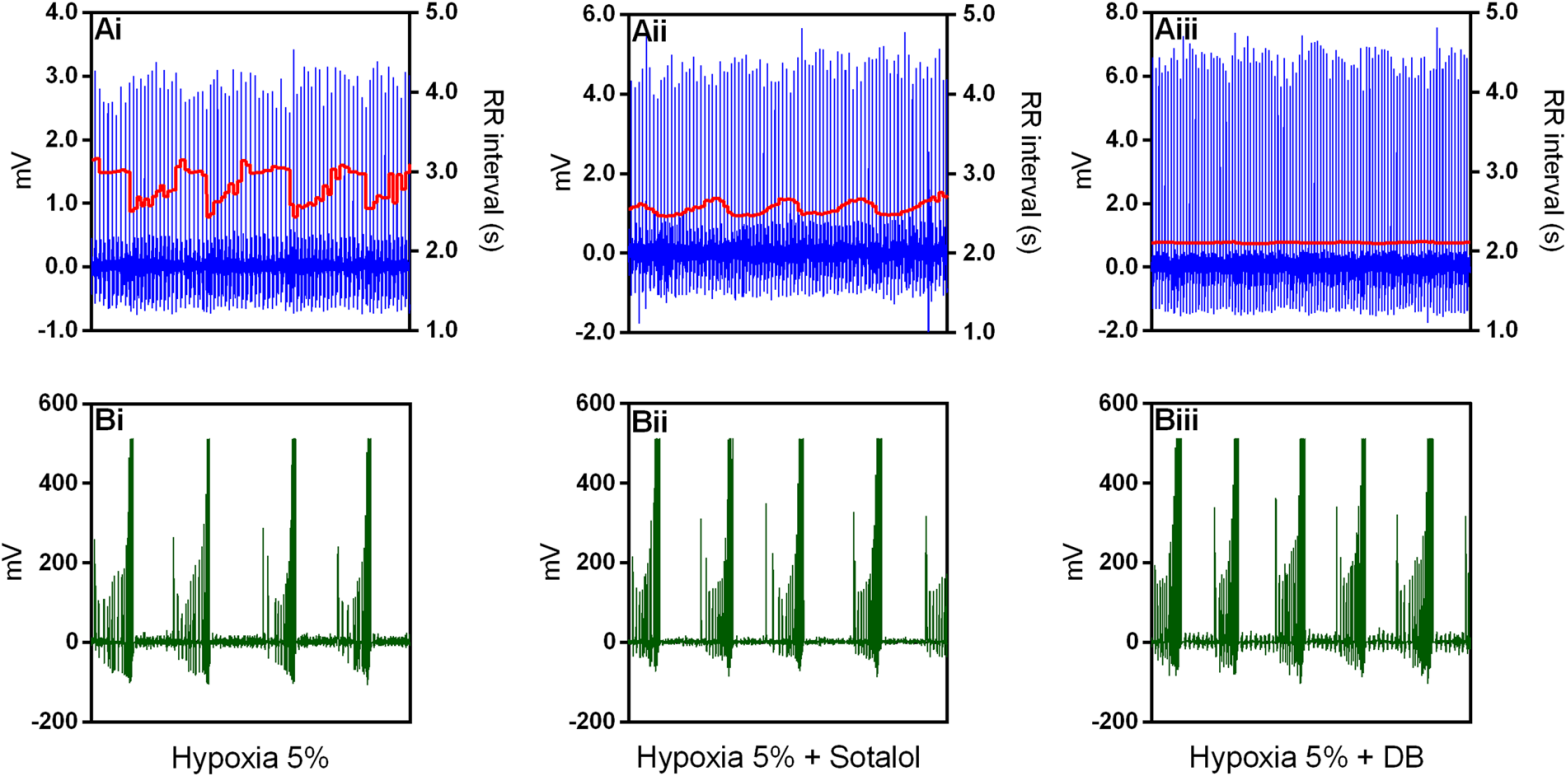
Representative recordings from one toad *Rhinella schneideri* (same as in Fig. 6) depicting raw electrocardiogram signals, the instantaneous RR interval and the associated lung inflation cycles (buccal pressure cannula) showing a cardiorespiratory interaction during hypoxia before (Ai and Bi), after β-adrenergic (Sotalol; Aii and Bii) and double autonomic blockade (DB) (Aiii and Biii). Note that the amplitude oscillations in the RR interval associated with respiratory cycles were diminished by the sympathetic blockade (sotalol) and the remaining oscillation was totally removed by the parasympathetic drug (atropine).

A representative from one individual is depicted in Figs 4 and 5 showing that atropine diminished the fundamental peak associated with lung inflation cycle during hypoxia. In addition, the remaining oscillatory component seen in the spectrum of the RR interval was further reduced, but not completely eliminated, after β-adrenergic blockade (Fig. 4). The oscillatory profile in instantaneous RR intervals related to each lung inflation cycle before and after selective autonomic blockade is depicted in the representative recordings in Fig. 5. An additional representative animal that received the reverse pharmacological treatment that consisted of injecting β-adrenergic antagonist before muscarinic blockade displayed a predominantly sympathetic modulation on the respiratory component exhibited in the spectrum of the RR interval (Fig. 6). The remaining respiratory-associated peak was completely abolished by atropine (Fig. 6A). The oscillatory profile in instantaneous RR intervals related to lung inflation cycles before and after selective autonomic blockade is depicted in the representative plots in Fig. 7.

## Discussion

This is the first study to demonstrate a common rhythm between heart rate oscillation and lung inflation cycles through power spectral analysis of the RR interval in a species of amphibian, the toad *Rhinella schneideri*. An oscillatory pattern in *f*_*H*_ recordings arises from each lung inflation cycle, a common respiratory profile exhibited under hypoxia exposure in anurans^13,15,16,20,21^. Thus, a marked peak in the spectrum of the RR intervals matching lung inflation cycle frequency was achieved by the presence of a constant breathing pattern driven by acute hypoxia exposure. The respiratory peak in the HRV was suppressed by autonomic blockade indicating that autonomic modulation plays a major role on such interactions. The expected mechanical effect of lung ventilation on the cardiovascular system is not essential for such cardiorespiratory interactions. Although *f*_*H*_ increased during lung inflation cycles under normoxic conditions, its scattered patterns prevented the observation of a well-marked ventilatory peak in the spectrum which covered a wide range of frequencies like previously seen in premature human neonates that exhibit an arrhythmic breathing patterns^2^.

The combination of instrumentation and short recovery periods would provide an experimental animal with an impaired autonomic balance^12,22^. We suggest such information be taken into account for any experimental design plans. Generally, lower heart rates are associated with higher parasympathetic tone relative to sympathetic influence on the heart^12,23^. Although surgically instrumented for cardiorespiratory measurements, our toads exhibited elevated vagal tone, the presence of a parasympathetic modulation for RSA and a very low sympathetic tone on the heart (Table 1; Fig. S1B; Supplementary information). Such results are in agreement with relatively low heart rates in our toads (~17 beats.min^−1^) and probably reflect minimal discomfort associated with both cannulae and electrodes, in addition to a long recovery time from surgical procedures (7 days). Higher heart rates have been reported in earlier studies for the same species (~29 bpm;^18,24^), in which maintenance of an intra-arterial catheter and short recovery time (*e*.*g*. ~24 hours) may contribute to a reduced vagal tone and/or enhanced sympathetic tone on the heart.

Cardiorespiratory-coupling originates from reciprocal interaction between cardiovascular and respiratory systems in vertebrates^1,2,18,25^. Simultaneous recordings of *f*_*H*_, pulmocutaneous blood flow (${\dot{Q}}_{{pc}}$) and ventilation in anuran amphibians have shown concurrent rises in *f*_*H*_ and ${\dot{Q}}_{{pc}}$ within each lung inflation cycle followed by a decrease in both parameters as the cycle ends^13,26^. Such interactions are clearly evident during hypoxia (F_I_O_2_: 0.05) in which a prevailing left-to-right shunt takes place accounting for the re-circulation of the pulmonary venous blood into the pulmonary circulation^13,27^ in these animals that have a three-chambered heart. Since blood pressure remains constant during hypoxia^13,24,27^, cardiac output increases mainly because of an increase in *f*_*H*_^13,24,27^ since stroke volume remains fairly constant^13^. Lung inflation cycles are accompanied by an almost two-fold increase in ${\dot{Q}}_{{pc}}$, while systemic blood flow tends to decrease^13,26^ or remains constant^27^. The redistribution of blood between systemic and pulmocutaneous circulation may be safeguarded by unloading of arterial baroreceptors that triggers marked increases in *f*_*H*_ in anurans in which sensitivity is primarily against hypotension^17,18,28^. Increases in *f*_*H*_, and maybe augmented vasomotor tone, within lung inflation cycles may occur in an attempt to avoid reductions in systemic vascular resistance thereby ensuring adequate oxygen delivery. Thus, it seems that sympathetic activity accounted for increases in *f*_*H*_ associated with lung inflation cycles, at least under acute hypoxia exposure in our toads.

Mammals RSA encompasses a beat-to-beat oscillation in the *f*_*H*_ signal matching ventilatory cycle^4^. Most studies on cardiorespiratory interactions in vertebrates describe the relationship between *f*_*H*_ and ventilation based on descriptive and/or averaged values comparisons^3,9–11,13^ and not their oscillatory component in the HRV signal. Resting South American rattlesnakes (*C*. *durissus terrificus*), a snake with a continuous breathing pattern, exhibit an oscillatory component at the frequency of ventilation similar to the mammalian RSA^12^. The lower that resting *f*_*H*_ is in rattlesnakes, the higher the respiratory component in the spectrum of the RR intervals^12^. A remarkably interesting feature in our toads is that the respiratory component in the HRV signal arose during hypoxia when sympathetic tone is augmented relative to resting animals reflecting an elevated *f*_*H*_ (see Fig. S1; Supplementary information). Besides a reduction trend in cholinergic tone under hypoxia, vagal influences on the heart still seem to have contributed to overall HRV in the toad *R*. *schneideri*. Particularly, neither single effects of cholinergic nor β-adrenergic blockade were able to totally abolish the persistence of some respiratory-related peaks in the spectrum of RR intervals. Despite that, only cholinergic blockade performed after β-adrenergic blockade abolished the remaining respiratory-peak (Table 1). Collectively, these data provide an interesting perspective for the underpinning source of autonomic modulation arising during hypoxia-induced cardiorespiratory interaction in amphibians. Similarly, in the cane toad *R*. *marina*, cholinergic blockade is capable of mimicking increases in ${\dot{Q}}_{{pc}}$ and *f*_*H*_ evoked by hypoxia, and most of these cardiovascular adjustments during bouts of ventilation represent a withdrawal of vagal tone on the heart and pulmocutaneous artery^13^. In decerebrated and unidirectionally ventilated *R*. *marina*, changes in ${\dot{Q}}_{{pc}}$ and *f*_*H*_ are also associated with the onset of spontaneous bouts of fictive breathing. Since phasic input from lung stretch receptors and chemoreceptors are absent, that association suggests a direct interaction within the central nervous system between cardiac vagal preganglionic neurons and pulmonary vagal preganglionic neurons^19^. An overlapping distribution between these two systems exist in the African clawed frog (*Xenopus laevis*) implying an important feed-forward mechanism for cardiorespiratory-coupling in this species^2,3^. Therefore, despite the fact that cardiac and pulmonary vagal motoneurons distribution has not yet been characterized in the toad *R*. *schneideri*, the overall variability in the *f*_*H*_ signal related to lung inflation cycles under hypoxia suggests an important central origin for interaction between cardiovascular and respiratory areas.

The physiological significance for cardiorespiratory interactions, such as RSA, remains unclear^7,29–32^. Nevertheless, Hayano *et al*.^7^ have suggested that the mammalian RSA is responsible for coupling pulmonary perfusion to ventilation thereby optimizing oxygen uptake and carbon dioxide removal. Consequently, resting animals and humans decrease the energy expenditure by suppressing unnecessary heartbeats during expiration^33^. While recent studies have examined RSA’s role in gas exchange improvement in humans, much remains unknown about cardiorespiratory interactions in lower vertebrates, and the phenomenon underlying its physiological relevance is yet to be explored. For instance, an artificial prevention of developing left-to-right shunts during lung ventilation does not impair resting gas exchange in *T*. *scripta*. This implies that cardiorespiratory interactions under resting conditions are not required for maintaining resting levels of pulmonary gas exchange while long-term effects may influence aerobic performance during diving^34^ or changes in body temperature.

Almost the entire HRV, especially the respiratory related-peak, was abolished after total autonomic blockade in our toads; however, a discrete ventilatory oscillation in *f*_*H*_ signal can be observed in some recordings (*e*.*g*. Fig. 5Aiii). A mechanical influence from lung inflation cycles may be responsible for this oscillatory pattern on *f*_*H*_ in which pressure changes in the intracoelomic cavity would thereby increase venous return and, thereby, *f*_*H*_ and cardiac output by the Frank-Starling mechanism^3,35^. In view of gas exchange improvement for cardiorespiratory interactions, mechanical effects during lung inflation cycles in *R*. *schneideri* may also contribute to cardiac output adjustments increasing pulmonary perfusion synchronously with serial increases in lung air volume. In summary, the cardiorespiratory interactions in anuran amphibians probably result from a combination of several factors like: (1) the central interaction between respiratory nuclei and cardiac vagal preganglionic neurons, (2) the afferent information from pulmonary stretch receptors and chemoreceptors that serves as a feedback mechanism releasing cardiac vagal tone on the heart and pulmonary artery, (3) the increases in *f*_*H*_ and, thereby, cardiac output by a Frank-Starling mechanism due to mechanical pressure changes in venous return imposed by lung ventilation, (4) and locally mediated responses by lung gas composition (CO_2_ and/or O_2_) affecting vascular tone (reviewed by Wang, *et al*.^3^). Therefore, their particular relevance and contribution may be emphasized under different environmental conditions.

## Conclusions

We demonstrated that the Cururu toad *R*. *schneideri* exhibits cardiorespiratory interactions that account for combined vagal tone release and augmented sympathetic modulation on the heart during the onset of lung inflation cycles. That is detectable when lung inflation frequency is high and regular after hypoxia stimulation. The increased sympathetic modulation related to the ventilatory peak in the RR interval spectrum may be a result of a feedback baroreceptor response mediated by sympathetic activation during the prevalence of left-to-right shunt within the undivided ventricle, counterbalancing reduced systemic blood flow and, thereby, hypotension in the systemic circuit. Baroreceptors in the aortic arch of the toad *R*. *schneideri* are exclusively involved in buffering unloaded pressures^36^ which entirely accounts for *f*_*H*_-baroreflex sensitivity in the toad *R*. *schneideri* ^17,18^. Therefore, the oscillatory peak in *f*_*H*_ variability produced by acute exposure to hypoxia in our toads likely reflects central interactions between respiratory and cardiovascular areas as previously suggested^19^. In addition, increased inputs from peripheral chemoreceptors due to low oxygen partial pressure is responsible for augmentation of the respiratory drive that may evoke a higher amplitude change in cardiovascular variables.

## Methods

### Animals

Cururu toads *Rhinella schneideri* (Werner, 1894), of both sexes (212 ± 23 g body mass) were collected in swampy areas in São Paulo State (Ribeirão Preto region and the city of Barbosa), Brazil (approximately 21° 10′ S and 47° 48′W, 21° 25′S and 49° 92′W, respectively) and transported to our laboratory at the Department of Animal Morphology and Physiology, UNESP, Jaboticabal, São Paulo, Brazil (approximately 21° 14′ S and 48° 17′W). All animals were maintained at 25 °C with a light:dark cycle of 12 h:12 h with free access to water from an artesian well. They were housed in containers containing coconut fibers as a substrate with tubes for hiding and were held under laboratory conditions at least three weeks before initiation of experimental protocols. Animals were fed two times a week with captive-bred Speckled cockroaches (*Nauphoeta cinerea*) that were dusted with calcium and vitamin D_3_. All experiments were performed from September to March which matches the activity season of this species^17,18^. Animals’ capture was approved by the Brazilian environmental agency (SISBIO-ICMBio/ no. 35484-1), and all experimental procedures were performed in accordance with guidelines and regulations approved by a regional Institutional Animal Care and Use Committee of São Paulo State University in Jaboticabal, Brazil (CEUA-FCAV-UNESP; Permit no. 017204/12).

### Surgical procedures

Animals were anesthetized by immersion in an aqueous 0.25% solution of 3-aminobenzoic acid ethyl ester (MS-222, Sigma-Aldrich, St. Louis, MO, USA), buffered to pH 7.7 with sodium bicarbonate for 10 min or more until toads lost their palpebral reflex. Electrocardiogram (ECG) recording electrodes were made out of three attached insulated stainless steel wires with 30-gauge hypodermic needles (with the beveled edge blunted) soldered onto one end of the wires and a tripolar socket soldered onto the other end of the wires. Two out of the three ECG electrodes were implanted subcutaneously over the scapular region and sutured in place. The third one was implanted close to the urostyle region. A polyethylene cannula (PE-50 connected to PE-10; Clay Adams, Parsippany, NJ, USA) filled with heparinized Ringer solution (100 i.u. mL^−1^ heparin) was inserted subcutaneously close to the right posterior lymph heart for drugs injections. An additional polyethylene cannula (PE-100, Clay Adams, Parsippany, NJ, USA) was inserted through a small hole drilled into the mouth to record changes in buccal pressure (respiratory signal). Electrodes and catheters were both short (approximately 14 cm), so they were gathered and taped together on the back of the animal to keep them from tangling during the 7 day recovery period. The day of experiments, extensors were connected to both the electrodes and the catheters to reach between the animal and the recording equipment. All surgical procedures lasted less than 30 min. Just after surgery, toads were treated with prophylactic antibiotic (enrofloxacin, Baytril®; 5.0 mg kg^−1^ s.c., Bayer S.A., São Paulo, SP, Brazil) and analgesic (Flunixina Meglumina, Banamine®; 1.0 mg kg^−1^ s.c., Schering-Plough, Kenilworth, NJ, USA) agents^37,38^. Each animal was allowed to recovery for 7 days in a temperature-controlled room at 25 °C and was then transferred to a temperature-controlled chamber 24 hours before the beginning of the experimental procedure.

ECG and breathing frequency were recorded simultaneously. Pressure changes in the buccal cavity were measured using a differential pressure transducer (MLT141Spirometer, ADInstruments, Sydney, Australia). ECG signal was amplified at 10,000X gain and filtered between 0.1 Hz and 20 kHz using a differential amplifier (A-M Systems, model 1700, Sequim, WA, USA). Both buccal cavity pressure and ECG signals were sampled at 1 kHz (sampling rate) using computer software LabChart v7.3.7 (ADInstruments, Sydney, Australia).

### Experimental protocol

Toads were housed in an acrylic water-jacketed chamber kept at the experimental temperature of 25 °C using a constant-temperature circulating water bath (9112A11B Programmable Model 9112 Refrigerated Circulator, PolyScience, Niles, IL, USA). Experiments began 8 days after surgery and 24 hours after placement of the animals into the experimental chamber. The experimental chamber was continuously flushed with humidified room air or humidified hypoxic gas mixture [(F_I_O_2_ = 0.05); see details below], and the temperature inside the chamber was continuously measured using a MLT415/M thermistor temperature sensor (ADInstruments, Sydney, Australia). During normoxic conditions, ECG was recorded continuously for 24 hours after which each animal had its autonomic nervous system selectively blocked with atropine sulfate and sotalol hydrochloride (Sigma-Aldrich, St. Louis, MO, USA) each dissolved in amphibian Ringer’s solution^18^. First, four out of nine animals received subcutaneous injections of the β-adrenergic antagonist sotalol hydrochloride (3.0 μg kg^−1^) followed by injection of the muscarinic receptor antagonist atropine sulfate (3.0 μg kg^−1^) one hour later. Pharmacological treatment orders were then reversed, and the other five animals were injected with atropine followed by sotalol subcutaneous injections. The effects of pharmacological treatments were quantified from data taken one hour after each drug injection. In order to evaluate cardiorespiratory interactions associated with hypoxia exposure^13^, another group of toads were exposed to hypoxia (F_I_O_2_ = 0.05) 8 days after surgical procedures. After one hour exposure under hypoxic conditions, seven out of thirteen animals were subcutaneously injected with sotalol hydrochloride (3.0 μg kg^−1^) followed by injections of atropine sulfate (3.0 μg kg^−1^). Pharmacological treatment orders were also reversed during hypoxic exposure with an additional six animals. The effects of pharmacological treatments were quantified from data taken one hour after each drug injection.

### Power spectral analysis of heart rate

In order to evaluate the sympathetic and parasympathetic cardiac modulation of the *f*_*H*_ fluctuations, we analyzed ECG traces consisting of 1,500-seconds periods containing no ectopic beats or artifacts. Data were obtained for periods of normoxia, hypoxia and selective autonomic blockade (atropine, sotalol, and double autonomic blockade). LabChart’s (v.7.3.7, ADInstruments, Sydney, Australia) algorithm that detects inflection points in ECG signals was used to generate beat-to-beat time series with cardiac interval values. Time domain analysis (standard deviation of RR intervals; SD_RR_) and frequency domain analysis (power spectral analysis; total power of the spectrum of RR intervals; TP_RR_) were carried out using CardioSeries software (v2.4, http://www.danielpenteado.com). Power spectral analysis of the RR interval series was performed by resampling beat-by-beat data points every 333 ms by cubic spline interpolation (3 Hz) containing one interpolated segment of 4096 points. Subsequently, a Hanning window was used to attenuate side effects, and a spectrum was calculated for the segment with a Fast Fourier Transform algorithm for discrete time series. Following data processing through a Fast Fourier Transform algorithm, the whole spectrum was integrated under normoxic conditions, while for hypoxic conditions, lung inflation cycle frequency histograms were taking into consideration for integration of the spectrum in specific frequency bands.

### Data analysis

The RR interval average, SD_RR_ (used as the simplest variable reflecting a measure of intra-individual variability), and the TP_RR_ were analyzed using a one-way repeated-measures ANOVA (factor: treatments) at several conditions: normoxia, hypoxia (F_I_O_2_ of 0.05), atropine, sotalol, and double autonomic blockade. Lung inflation cycles were presented as frequency distribution histograms binned into 0.001 Hz width bands for comparison purposes with the spectra of the RR interval. In all ANOVA analyses the differences among means were further assessed by Tukey’s post hoc tests and were considered significant when *P* < 0.05. Data were tested for unequal variance and normality, and, when the data did not follow a normal distribution, logarithmic transformations were performed.

## Electronic supplementary material

Supporting File
