# Supplementary material for: Analysis of the respiratory component of heart rate variability in the Cururu toad Rhinella schneideri
Source: Sci Rep. 2017 Nov 23;7:16119. doi: 10.1038/s41598-017-16350-0 (PMC5701079; doi:10.1038/s41598-017-16350-0)
Supplement: Supplementary file 1 — Supporting File [file 41598_2017_16350_MOESM1_ESM.pdf]

Supporting File

Analysis of the respiratory component of heart rate variability in the Cururu toad *Rhinella schneideri*

Lucas A. Zena[1,4]*, Cléo A. C. Leite[2,4], Leonardo S. Longhini[1,4], Daniel P. M. Dias[5], Glauber S. F. da Silva[1,4], Lynn K. Hartzler[3], Luciane H. Gargaglioni[1,4], Kênia C. Bícego[1,4]

[1]Department of Animal Morphology and Physiology, College of Agricultural and Veterinary Sciences, São Paulo State University, 14884-900, Jaboticabal, São Paulo, Brazil.

[2]Department of Physiological Sciences, Federal University of São Carlos, São Paulo, Brazil.

[3]Department of Biological Sciences, Wright State University, Dayton, Ohio, USA.

[4]National Institute of Science and Technology in Comparative Physiology (INCT Fisiologia Comparada), Brazil

[5]Department of Physiology, Ribeirão Preto Medical School, University of São Paulo, São Paulo, Brazil

Figure Captions

Fig. S1. Overall variability of the RR interval and autonomic tone in the toad *Rhinella schneideri.* (A) 250 consecutive heartbeats under normoxic baseline conditions (blue circles; N = 9), after selective muscarinic (atropine; red circles; N = 5), β-adrenergic blockade (sotalol; green circles; N = 4) and double autonomic blockade (open circles; N = 9). (B) Relative cholinergic and adrenergic tone in the toads calculated as proposed by Altimiras *et al.* (1997) under normoxic [F_IO_2: 0.21 (pharmacological blockade performed with atropine sulfate (3.0 μg kg^{-1}) followed by sotalol hydrochloride (3.0 μg kg^{-1})], and under hypoxic [F_IO_2: 0.05 (pharmacological blockade performed with sotalol hydrochloride (3.0 μg kg^{-1}) followed by atropine sulfate (3.0 μg kg^{-1})]. The autonomic tonus depends on the amount of O_2 present in the inspired air, and the prevailing cholinergic tone in normoxia gives place to an elevated adrenergic tone in hypoxia (interaction between F_IO_2 and autonomic tonus factors: $F_{(1,22)} = 7.804$; $P = 0.019$; Tukey's test). Data are means±s.e.m.

Fig. S2. Spectrum of the RR interval and associated histograms for lung inflation cycles in the toad *Rhinella schneideri* under hypoxic conditions. Individual spectrum plots for four different toads acutely exposed to hypoxia (F_IO_2: 0.05). The expressed cardiorespiratory interaction evoked during hypoxia is shown as a main peak in the spectrum of the RR interval

plots (A, C, E and G) that matches lung inflation cycles (represented as a frequency distribution histograms binned into 0.001 Hz width band; B, D, F and H). Note that the main peak in the spectrum plots shifts to higher or lower frequency in the *x-axis* according to the respiratory response to each toad to hypoxia.

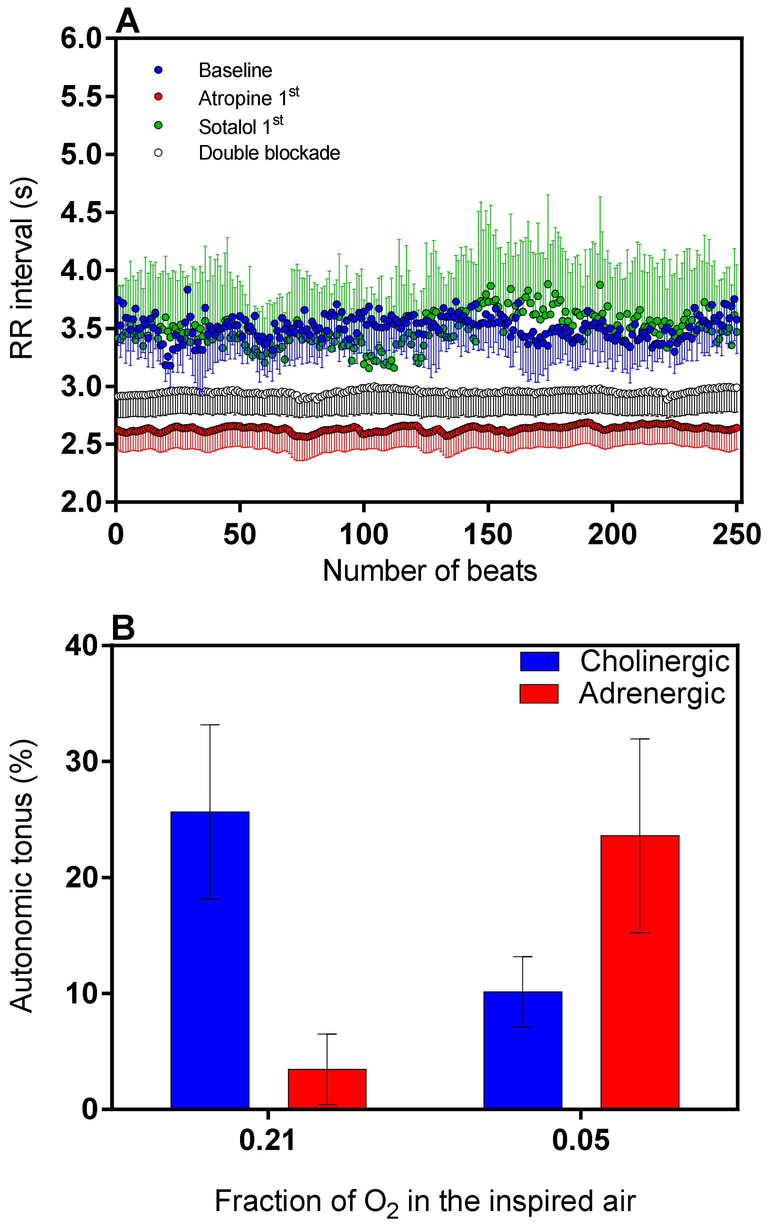

Fig. S1

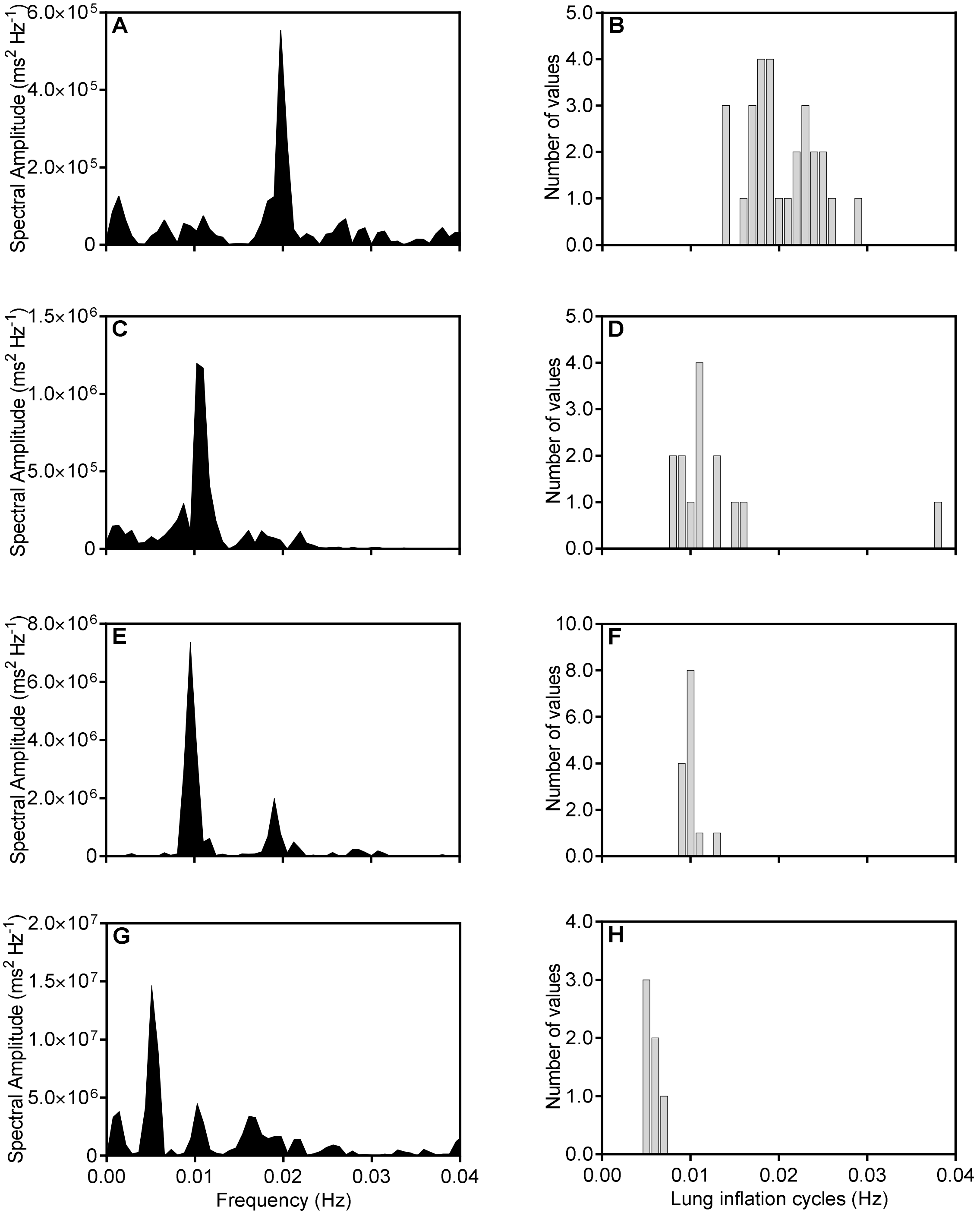

A
B
C
D
E
F
G
H
Spectral Amplitude (ms^2 Hz^{-1})
Number of values
6.0×10^5
4.0×10^5
2.0×10^5
1.5×10^6
1.0×10^6
5.0×10^5
8.0×10^6
6.0×10^6
4.0×10^6
2.0×10^6
2.0×10^7
1.5×10^7
1.0×10^7
5.0×10^6
0
5.0
4.0
3.0
2.0
1.0
0.0
10.0
8.0
6.0
0.00
0.01
0.02
0.03
0.04
Frequency (Hz)
Lung inflation cycles (Hz)

Fig. S2
